# *Aquilaria crassna* Leaf Extract Ameliorates Glucose-Induced Neurotoxicity In Vitro and Improves Lifespan in *Caenorhabditis elegans*

**DOI:** 10.3390/nu14173668

**Published:** 2022-09-05

**Authors:** Nattaporn Pattarachotanant, Nilubon Sornkaew, Watis Warayanon, Panthakarn Rangsinth, Chanin Sillapachaiyaporn, Wudtipong Vongthip, Siriporn Chuchawankul, Anchalee Prasansuklab, Tewin Tencomnao

**Affiliations:** 1 Natural Products for Neuroprotection and Anti-Ageing (Neur-Age Natura) Research Unit, Faculty of Allied Health Sciences, Chulalongkorn University, Bangkok 10330, Thailand; 2 Department of Clinical Chemistry, Faculty of Allied Health Sciences, Chulalongkorn University, Bangkok 10330, Thailand; 3 Department of Transfusion Medicine and Clinical Microbiology, Faculty of Allied Health Sciences, Chulalongkorn University, Bangkok 10330, Thailand; 4 Program in Clinical Biochemistry and Molecular Medicine, Department of Clinical Chemistry, Faculty of Allied Health Sciences, Chulalongkorn University, Bangkok 10330, Thailand; 5 College of Public Health Sciences, Chulalongkorn University, Bangkok 10330, Thailand

**Keywords:** neurite outgrowth, GAP-43, Teneurin-4, Cyclin D1, SIRT1, *daf-16*, *aqp-1*

## Abstract

Hyperglycemia is one of the important causes of neurodegenerative disorders and aging. *Aquilaria crassna* Pierre ex Lec (AC) has been widely used to relieve various health ailments. However, the neuroprotective and anti-aging effects against high glucose induction have not been investigated. This study aimed to investigate the effects of hexane extract of AC leaves (ACH) in vitro using human neuroblastoma SH-SY5Y cells and in vivo using nematode *Caenorhabditis elegans*. SH-SY5Y cells and *C. elegans* were pre-exposed with high glucose, followed by ACH treatment. To investigate neuroprotective activities, neurite outgrowth and cell cycle progression were determined in SH-SY5Y cells. In addition, *C. elegans* was used to determine ACH effects on antioxidant activity, longevity, and healthspan. In addition, ACH phytochemicals were analyzed and the possible active compounds were identified using a molecular docking study. ACH exerted neuroprotective effects by inducing neurite outgrowth via upregulating growth-associated protein 43 and teneurin-4 expression and normalizing cell cycle progression through the regulation of cyclin D1 and SIRT1 expression. Furthermore, ACH prolonged lifespan, improved body size, body length, and brood size, and reduced intracellular ROS accumulation in high glucose-induced *C. elegans* via the activation of gene expression in the DAF-16/FoxO pathway. Finally, phytochemicals of ACH were analyzed and revealed that β-sitosterol and stigmasterol were the possible active constituents in inhibiting insulin-like growth factor 1 receptor (IGFR). The results of this study establish ACH as an alternative medicine to defend against high glucose effects on neurotoxicity and aging.

## 1. Introduction

Neurodegenerative disease (ND) comprises a group of neuronal disorders such as Alzheimer’s disease [1], Parkinson’s disease (PD) [2], Huntington’s disease, etc. ND results in the damage of neuronal structure and function and the inhibition of neuronal differentiation. Hyperglycemia is a key factor causing neuronal damage and neuronal differentiation inhibition [1,3]. Previous studies have indicated that high glucose concentration induced SH-SY5Y neuronal cell damage through the induction of cell cycle arrest via the downregulation of cyclin D1, phospho-cell division cycle 2 (pcdc2), and phospho-Retinoblastoma (pRb) proteins. Moreover, high glucose concentration reduced the healthspan and lifespan of *Caenorhabditis elegans* in a round worm model via the increase of reactive oxygen species (ROS) formation and advanced glycation end product (AGEs) modification of mitochondrial proteins in a daf-2 independent manner [4].

*Aquilaria crassna* Pierre ex Lec (AC) belongs to the Thymelaeaceae family and is well known as agarwood. AC is a fragrant wood that has many ties with religious history, rituals, and ceremonies [5]. It is widely used as an ingredient in food, including medicinal wine in traditional Chinese and Korean medicines [6], biscuits, herbal soups, and instant noodles [7]. For pharmacological activities, plant materials from AC have been reported to exert different bioactivities, including the inhibition of inflammatory cytokines (TNF-α and IL-1α) [8] as well as anti-ischemic (cardioprotective) [9,10], antianaphylaxis [11], hepatoprotective [12], anti-cancer [13], and antioxidant [13,14] properties.

Previous studies have shown that the extract of AC leaf demonstrates neuroprotection and neuritogenesis in vitro [15] as well as antihyperglycemia and glucose-uptake enhancement activities in streptozotocin-induced diabetic rats [16]. Nevertheless, the pharmacological activities and underlying mechanisms of AC against hyperglycemia-associated neurodegenerative diseases and the effect on healthspan and lifespan need to be clarified.

The present study aimed to investigate the neuroprotective and anti-aging effects of hexane extract AC (ACH) and its underlying mechanisms on high glucose-induced neurotoxicity using human neuroblastoma cells (SH-SY5Y) and high glucose-induced life span and healthspan reduction in a *C. elegans* model. The neuroprotective properties of ACH were evaluated through the ability of this extract to exert neuritogenesis potential, including neurite outgrowth formation, and to induce cell cycle normalization. The anti-aging effect of ACH in *C. elegans* was provided by the ability to prolong life span and improve body size, body length, and brood size. In addition, possible active phytochemicals in AC were also analyzed by in silico molecular docking, gas chromatography-mass spectrometry/mass spectrometry (GC-MS/MS), reverse-phase high-pressure liquid chromatography (RP-HPLC), and nuclear magnetic resonance (NMR). Therefore, this experiment provided experimental evidence of the applications of ACH in the prevention or treatment of neurodegeneration and aging associated with high glucose conditions.

## 2. Materials and Methods

### 2.1. Chemicals and Reagents

Dimethyl sulfoxide (DMSO), hexane, and ethanol were purchased from Merck KGaA (Darmstadt, Germany). Phenylmethyl sulphonyl fluoride (PMSF) was purchased from United States Biological (Cleveland, OH, USA). Kodak processing chemicals were used for autoradiography films. The Amersham ECL select Western blotting detection reagent was purchased from Merck KGaA (Darmstadt, Germany). Dulbecco’s modified Eagle medium (DMEM)/low glucose, fetal bovine serum (FBS), and penicillin-streptomycin solution (10,000 units/mL of penicillin and 10,000 μg/mL of streptomycin) were purchased from Thermo Scientific HyClone (Logan, UT, USA). A solution of 30% acrylamide/bis-acrylamide (37.5:1) was purchased from Bio-Rad Laboratories (Hercules, CA, USA). Ammonium persulfate (APS) was purchased from Millipore Sigma (Burlington, MA, USA). The monoclonal rabbit Cyclin D1 (92G2, cat#2978) and β actin (13E5, cat#4970) were purchased from Cell Signaling Technology (Beverly, MA, USA). The monoclonal mouse SIRT1 (B-7, sc-74465) was purchased from Santa Cruz Biotechnology (Dallas, TX, USA). The polyclonal sheep IgG Teneurin-4 (AF6320) and sheep IgG HRP-conjugated antibody (HAF016) were from R&D systems, Inc. (Minneapolis, MN, USA) and the monoclonal rabbit GAP43 (ab75810) antibody from Abcam (Cambridge, UK).

Propidium iodide (PI) was purchased from Biolegend (San Diego, CA, USA). 3-(4,5-dimethylthiazol-2-yl)-2,5-diphenyltetrazoliumbromide (MTT) was purchased from Bio Basic Inc. (Markham, ON, Canada). Trizol reagent was purchased from Thermo Fisher Scientific (Waltham, MA, USA).

### 2.2. Plant Extraction

Leaves of *Aquilaria crassna* (agarwood) were dried under shade and ground into fine powder. Successive extraction was performed by the maceration method using hexane (40 g of dried plant: 400 mL of solvent) at room temperature for 72 h. The supernatant was collected, filtrated, and evaporated by the rotary evaporator at 45 °C. The phytochemical constituents were stabilized by keeping dried crude extract at −20 °C and protected from light exposure The extract was dissolved in DMSO as stock solution (100 mg/mL), passed through a 0.2 μm filter, and stored at −20 °C until use. Hexane extraction of *Aquilaria crassna* was designated as ACH.

### 2.3. Cell Line

SH-SY5Y cells were purchased from a cell line service (Heidelberg, Germany) and were cultured in DMEM/low glucose (HyClone, Logan, UT, USA) containing 10% FBS and antibiotics including 100 U/mL penicillin and 100 μg/mL streptomycin at 37 °C in a humidified atmosphere at 5% CO_2_.

### 2.4. Antioxidant Determination

These assays were modified for a microplate format, as previously described [17,18].

#### 2.4.1. Folin–Ciocalteu Phenol Assay (FCP)

The extracts (50 μL) and 10% Folin–Ciocalteu phenol reagent (50 μL) were mixed and incubated in the dark at room temperature (RT) for 30 min. A sodium carbonate (Na_2_CO_3_) solution (50 μL) was added, mixed, and incubated in the dark at RT for 20 min. Reaction absorbance was measured using the Enspire^®^ Multimode Plate Reader (Perkin-Elmer) at 760 nm. Gallic acid was used as the standard. The amount of phenolic compound was in a Gallic acid equivalent (GE) mg/g of dry weight.

#### 2.4.2. Determination of Total Flavonoid

The extracts (50 μL) were mixed with the solution containing 150 μL of ethanol, 10 μL of 1M Sodium acetate (NaOAc), and 10 μL of Aluminum Chloride (AlCl_3_). The mixture was incubated in the dark at RT for 40 min and measured at 415 nm. Quercetin was used as the standard. The content of flavonoid was in Quercetin equivalent (QE) mg/g of dry weight.

#### 2.4.3. Radical Scavenging Activity Assays

First, working 2,2′-azino-bis (3-ethylbenzthiazoline-6-sulphonic acid) (ABTS•^+^) (OD_734_ = 0.7–0.8) solution was prepared by the addition of 2.45 mM K_2_S_2_O_8_ to 7 mM ABTS (ratio 2:3) and incubated at 4 °C for 16–18 h. In addition, 0.2 mM of 2,2-diphenyl-1-picryl-hydrazyl-hydrate (DPPH•) (OD_517_ = 0.8–0.9) was diluted in ethanol.

The extract (1 mg/mL) was reacted with DPPH• or ABTS•^+^ and incubated at RT for 15 or 30 min, respectively. Absorbance was measured at 517 nm and 734 nm, respectively. Ascorbic acid or Vitamin C was used as the standard for both assays. The antioxidant capacity had Vitamin C equivalent antioxidant capacity (VCEAC) in mg/g of dry weight.

### 2.5. MTT Assay

To verify which ACH concentrations were nontoxic, SH-SY5Y cells were treated with ACH extract at the concentrations of 10, 25, 50 and 100 μg/mL for 24 h. Then, 20 μL of 5 mg/mL MTT was added to each well and incubated at 37 °C for 4 h. After incubation, 10% SDS in 0.01 NHCl (50 μL) was added and incubated overnight to dissolve the formazan crystal. Finally, the absorbance at 570 nm with a spectrometer was performed. The percentage of cell viability compared to the control group was a nontoxic concentration [19].

### 2.6. Intracellular Reactive Oxygen Species (ROS) Assay

For cell culture, cells were seeded at 10,000 cells/well in 96-well black plates with a clear bottom and cultured at 37 °C for 24 h. Having been incubated, cells were treated with high glucose (50 mM) for 48 h. Subsequently, cells were post-treated with ACH extract at the concentration of 10, 25, 50, and 100 μg/mL for 24 h. The ROS level was measured by following the published protocol [20]. After treatment, 5 μM (diluted in serum-free culture medium) of non-fluorescent 2′,7′-dichloro-dihydrofluorescein diacetate (H_2_DCFDA) was loaded, incubated for 45 min, and then washed three times with PBS. The fluorescence was measured with an excitation wavelength of 485 nm and an emission wavelength of 535 nm.

### 2.7. Neurite Outgrowth Assay and Scoring of Neurite Length and Neurite-Bearing Cells

SH-SY5Y cells were cultured in 10% FBS culture medium for 24 h and induced the differentiation with 1% FBS culture medium and 10 μM retinoic acid (RA) for 24 h. During RA-induced differentiation, the effect of high glucose (50 mM) on neurite outgrowth inhibition was observed following addition for 48 h. Likewise, the protective effect on high glucose-induced neurite outgrowth inhibition of ACH was detected after cells were treated with ACH (10 and 50 μg/mL) for 24 h.

Cells were imaged by a differential interference contrast (DIC) microscope for 3 independent experiments. For neurite scoring, a cell that had at least 1 neurite extension longer than the diameter of its cell body was scored as a neurite-bearing cell. Cell clusters contained more than 5 cells were excluded [21]. Otherwise, the neurite length was obtained by the measurement of the longest neurite in each image using the software Motic Images Plus 3.0 [22].

### 2.8. Cell Cycle Analysis

SH-SY5Y cells were treated with 50 mM glucose for 48 h. Having been incubated, cells were post-treated with ACH for 24 h. Then, cells were fixed with precooled absolute ethanol at −20 °C for at least 2 h and stained with propidium iodide containing RNase A at 37 °C for 15 min in the dark [20]. Cell cycle progression was analyzed using a BD FACSCalibur flow cytometer (BD Biosciences, San Jose, CA, USA).

### 2.9. Western Blot Analysis

To identify the pathway through which ACH extract attenuates high glucose-induced neurite outgrowth inhibition and cell cycle progression delay, SH-SY5Y cells were treated as explained earlier. Total protein was extracted using NP-40 lysis buffer. Western blotting was performed using the standard experimental procedure [20] with primary antibodies, including cyclin D1 (1:2000), SIRT1 (1:5000), GAP-43 (1:5000), Teneurin-4 (1:2000), and β-actin (1:5000). Chemiluminescence detection reagent was used to develop HRP-linked secondary antibody bands. These bands were obtained using ImageJ software.

### 2.10. C. elegans Strain, Maintenance, Synchronization, and Treatment

Wild type *C. elegans* or Bristol N2 were cultivated on nematode growth medium (NGM) agar and maintained at 20 °C on living *Escherichia coli* (OP50) with an OD_600_ of 1.5. However, in the high glucose-treated experiment using dead bacteria, *E. coli* OP50 were incubated at 65 °C for at least 30 min to stop bacteria growth [23].

*C. elegans* and *E. coli* OP50 were obtained from the Caenorhabditis Genetics Center (University of Minnesota, Twin Cities, MN, USA). To exclude the antimicrobial activity of ACH against *E. coli* OP50, an agar diffusion test was conducted.

Age synchronization of the worms was as previously described [18]. For all experiments, N2 synchronized L1 larvae were kept on NGM agar plates containing dead *E. coli* OP50 (OD_600_ = 1.5) and divided into 4 groups. The first was treated with 0.1% DMSO (solvent control group). The second was treated with 50 mM glucose. Groups three and four were co-treated with 50 mM glucose and ACH at the concentrations of 10 and 50 μg/mL, respectively.

### 2.11. Brood Size, Body Length, and Body Size

These experiments were modified from the published protocol [18,24]. For brood size assay, L1 larvae stage worms in each group containing *E. coli* OP50 and treatment were cultured at 20 °C. After 48 h, L4 larvae was individually sorted and transferred to the new NGM containing *E. coli* OP50 and the treatment. L4 larvae were allowed to grow into adults and lay eggs. Under a dissecting microscope, eggs were observed and counted. While waiting for the adult worms to stop laying eggs, eggs were separated from adult worms every day.

For body length and size assay, at least 20 adult day 1 worms in each group were imaged by a 10× objective lens of a bright-field microscope. The body length and size of adult worms was analyzed by the software Motic Images Plus 3.0.

### 2.12. Intracellular ROS Accumulation

N2 synchronized L1 larvae were treated and cultured. At adult day 1, worms were stained with 50 μM H_2_DCFDA and incubated away from light for 45 min to 1 h at 20 °C. Having been stained, worms were washed with M9 and mounted on a glass slide. Next, 10 mM sodium azide was added to paralyze the worms [18]. At least 30 worms were photographed using a confocal microscope (Olympus FluoView FV10i). Fluorescent intensity was measured and analyzed using the software ImageJ from National Institutes of Health (Bethesda, MD, USA).

### 2.13. Lifespan Assay

N2 synchronized L1 larvae were cultured on different NGM containing *E. coli* OP50 treated with 0.1% DMSO, 50 mM glucose, or ACH. At the L4 stage, worms were transferred to new NGM every day to separate them from their new population and to avoid starvation.

### 2.14. RT-qPCR

To identify the pathway through which ACH extract attenuates high glucose-induced lifespan and healthspan reduction, L1 larvae stage worms were treated and cultured for 72 h. Further, adult day 1 worms were collected and RNA was extracted by Trizol reagent following the manufacturer’s instructions. Reverse transcription followed the recommended protocol of Maxime RT PreMix Kit. The qPCR was accomplished in CFX Real time PCR, and the measured fluorescent signals indicated the PCR results. qPCR conditions were 95 °C for 15 min, denaturation at 95 °C for 15 s for 45 cycles, and primer annealing/extension at 55 °C for 30 s. The gene-specific primers were *daf-16*, *sod-3*, *aqp-1*, and *act-1*. The sequences of these RNA primers were shown in Table 1.

### 2.15. Phytochemical Constituent Analysis of Extracts by Gas Chromatograph-Mass Spectrometer/Mass Spectrometer (GC-MS/MS) Analysis

The components of ACH were analyzed at the Scientific and Technological Research Equipment Center (STREC) (Chulalongkorn University, Bangkok, Thailand), according to the standard procedure [20]. The GC-MS Triple Quad system was an Agilent 7890B GC system coupled with an Agilent 7000C MS and a capillary column characterized as HP-5MS 5% Phenyl Methyl Siloxane, length 30 m, i.d. 0.25 mm, and phase thickness 0.25 μm. The GC was operated with helium as the carrier gas at the rate of 1 mL/min. The inlet had a temperature of 250 °C, pressure set to 8.2317 psi, and 1 μL injection. The GC oven was initially at 60 °C before rising to 300 °C with a linear gradient of 3 °C/min and kept at 300 °C for 5 min. The total run time was 85 min. Approximately 20 mg of the extract was dissolved in 1 mL of hexane.

The obtained spectra were used to calculate the retention indices and authentic mass spectra data, and these were compared with those stored in the NIST Mass Spectrometry Data Center to identify phytochemical constituents. With the purpose of obtaining the retention indices, a series of *n*-alkane calibration standards (C8-C40) was run in the same condition and calculated with the Kovats Index [25,26].

### 2.16. Plant Extract Isolation and Identification

ACH extract was isolated by column chromatography using the condition hexane:ethyl acetate (EtOAc) (90:10). The eluates were examined by thin layer chromatography (TLC), and combined fractions were obtained. Interesting fractions were subjected to column chromatography on silica gel using hexane:EtOAc (90:10) to isolate their compounds, and their structures were elucidated by using ^1^H-NMR.

^1^H-NMR were recorded on a Bruker AVANCE 400 FT-NMR spectrometer operating at 400 (^1^H) MHz. Column chromatography was carried out using Merck silica gel 60 (finer than 0.063 mm). For TLC, Merck precoated silica gel 60 F_254_ plates were used. Spots on TLC were detected under UV light and by spraying with anisaldehyde-H_2_SO_4_ reagent followed by heating.

### 2.17. Reverse-Phase High-Pressure Liquid Chromatography (RP-HPLC)

The presence of active components in fractions of ACH was analyzed using reverse-phase high-performance liquid chromatography (RP–HPLC). The HPLC system consisted of a binary solvent delivery pump (Scientific, Spectra SYSTEM™ P1000 isocratic pump properl), column compartment, and Photo Diode Array detector. A Shodex HPLC Column Silica C18-4E, 120 Å, 5 μm, 4.6 × 250 mm was used as the stationary phase, and the peaks were obtained using Lab solutions software. The separation of compounds was performed by the isocratic mobile phase using methanol:acetonitrile in the ratio (90:10) with a flow rate of 1.0 mL min^−1^, and the column was set at ambient temperature. The samples and standards were injected using SGE syringe into the injection loop and were detected at 208 nm. The presence of stigmasterol at 28.697 min and β-sitosterol at 32.447 min were identified by comparing its retention time with ACH.

### 2.18. Molecular Docking

#### 2.18.1. Ligand Preparation

All chemical structures of the phytochemicals were obtained from the PubChem database. We minimized the energy, cleaned the geometry, and generated the PDB format of all chemical structures using BIOVIA Discovery Studio Visualizer (version 20.1) (San Diego, CA, USA). Next, we converted the file format to the protein databank, partial charge (Q), and atom type (T) or PDBQT using AutoDockTools-1.5.6 software (The Scripps Research Institute, San Diego, CA, USA) [27].

#### 2.18.2. Protein Preparation

The X-ray crystallographic structures of insulin-like growth factor 1 receptor (IGFR) (PDB ID: 5FXS) [28] were retrieved from the RCSB Protein Data Bank. Protein structures were processed using the Prepare Protein Set Up in AutoDockTools (version 1.5.6) software. The complex structure composed of all water molecules and the original ligand was excluded. Next, the complex protein structure was added with all the missing hydrogens and Kollman charges and then converted to PDBQT file format as the inputs for the docking study [29].

#### 2.18.3. Molecular Docking

The docking analyses were performed according to the previous report [29]. In brief, the AutoDock 4.2 software package supported by AutodockTools 1.5.6 was used. The Lamarckian Genetic Algorithm with default parameters was used to perform the protein–ligand interaction studies, and the results were further visualized using the Discovery Studio Visualizer (BIOVIA, San Diego, CA, USA).

### 2.19. Statistical Analysis

All data were presented as the mean ± standard error mean (SEM). Means were from at least 3 independent experiments. All data were analyzed by a one-way analysis of variance (ANOVA) followed by a post hoc Tukey test (*p*-value < 0.05) using low glucose-treated cells as the control group.

## 3. Results

### 3.1. Antioxidant Properties and Total Phenolic and Flavonoid Contents

The ACH extraction yield was 2.98% (*w*/*w*). The phenolic and flavonoid analysis showed that ACH contained 6.75 ± 1.29 mg GAE/g dry weight for total phenolic content and 2.49 ± 1.51 mg QE/g dry weight for total flavonoid content. The antioxidant activity was tested using DPPH and ABTS assays. The data of antioxidant capacity were stated in mg of vitamin C equivalent antioxidant capacity (VCEAC)/g dry weight. ACH had antioxidant capacities of 4.96 ± 0.49 mg VCEAC/g dry weight in DPPH and 3.07 ± 0.98 mg VCEAC/g dry weight in ABTS. Moreover, ACH possessed the free radical scavenging capacities of 3.55 ± 1.64% and 5.07 ± 0.73% against DPPH and ABTS radicals, respectively.

### 3.2. Effects of ACH on Cell Viability and High Glucose-Induced Reactive Oxygen Species (ROS)

**Figure 1:**
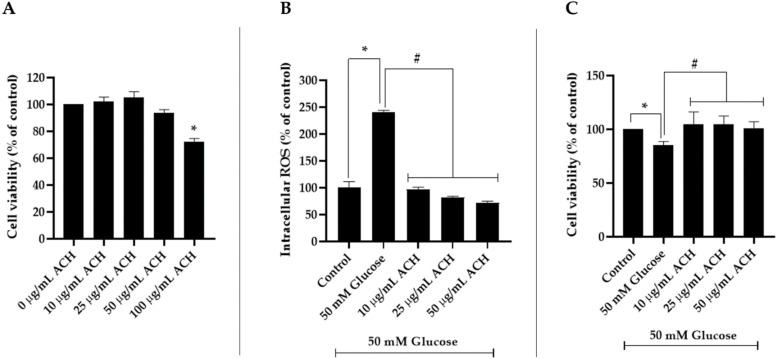
The effect of different concentrations of ACH on SH-SY5Y cell viability (**A**). MTT assay was used to clarify cell viability. The effect of ACH extracts on high glucose-induced intracellular ROS accumulation was performed using H2DCFDA assay, and the relative intracellular ROS accumulation level is shown in (**B**). MTT assay showed cell viability in cells pre-exposed with high glucose and followed by ACH treatment (**C**). Data are presented as the means ± SEM, * *p* < 0.05 vs. control and ^#^ *p* < 0.05 vs. 50 mM glucose.

We found that ACH extract at the concentrations of 10–50 μg/mL had no significant effect on cell viability (Figure 1A). Next, to examine the effects of ACH on high glucose-induced intracellular ROS, the experiment was modified from the previous protocol to investigate the treatment of SH-SY5Y cells with 50 mM glucose alone for 48 h. After high glucose treatment, cells were added with the extracts and cultured for 24 h. The H2DCFDA assay revealed that 10–50 μg/mL of concentrations showed a significant reducing effect on the intracellular ROS accumulation (^#^ *p* < 0.05 vs. 50 mM glucose) (Figure 1B). Moreover, the effects of ACH on high glucose-induced cytotoxicity were also examined. In the MTT assay, SH-SY5Y cells were pre-exposed with 50 mM glucose, followed by ACH treatment. As shown in Figure 1C, we found that glucose at the concentration of 50 mM could significantly decrease cell viability, while ACH (10–50 μg/mL) treatment alleviated the effect of glucose-induced cytotoxicity and significantly increased cell viability.

### 3.3. Effects of ACH on Neurite Outgrowth

**Figure 2:**
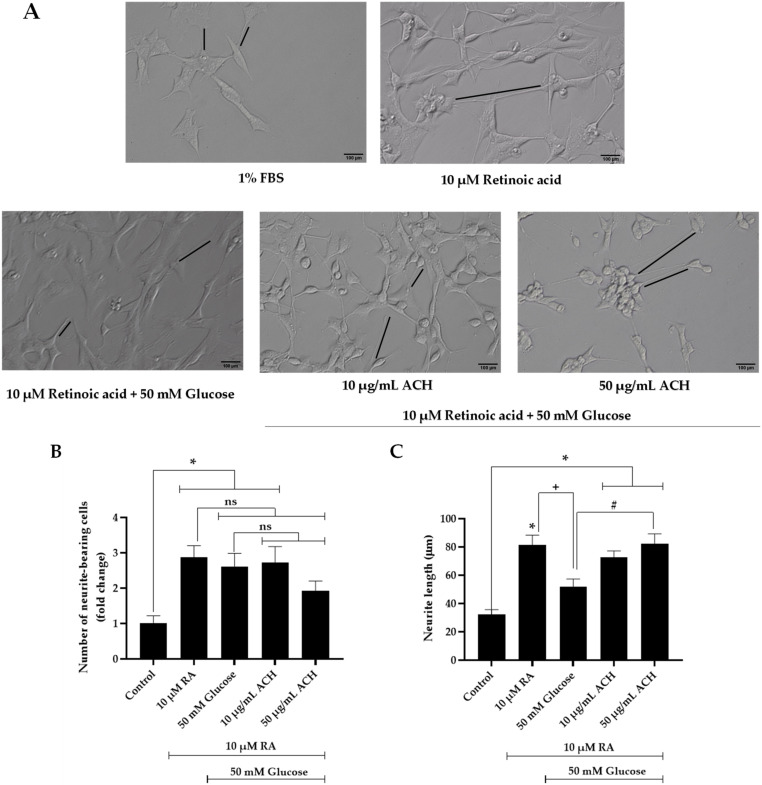

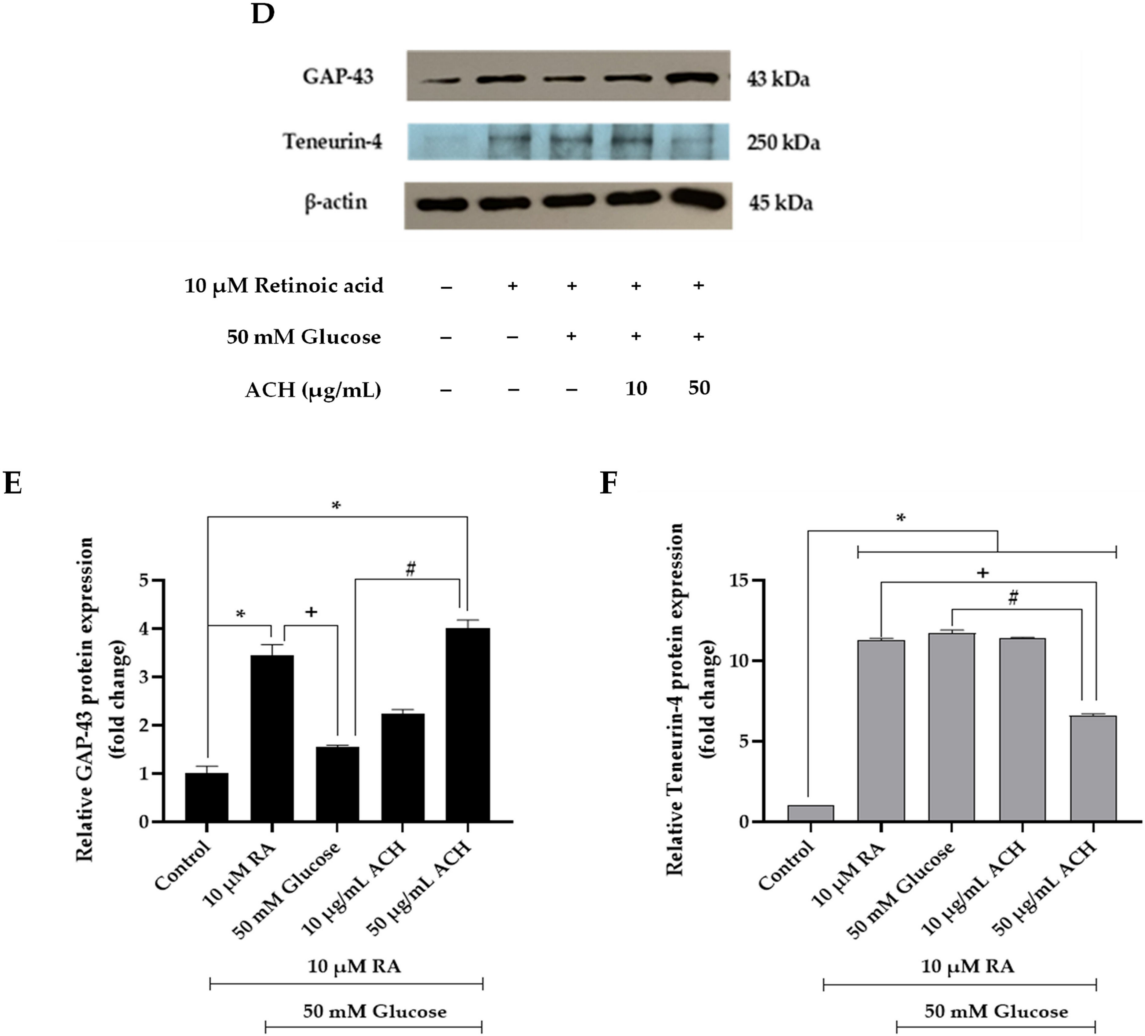
The neuroprotective effect of ACH on neuronal differentiation. The neurite outgrowth process was observed under a differential interference contrast (DIC) microscope at 10× magnification (black lines represented the measurement of neurite outgrowth) (**A**). The number of bearing cells and neurite length are shown in (**B**,**C**), respectively. GAP-43 and Teneurin-4 expression is shown in a representative Western blot (**D**). Normalized values of both GAP-43 and Teneurin-4 against β-actin (**E**,**F**). All data are presented as the mean ± SEM, * *p* < 0.05 vs. control; ^+^ *p* < 0.05 vs. 10 μM RA; ^#^ *p* < 0.05 vs. 50 mM glucose; ns = not significant.

Neuronal differentiation is one of the neuroprotective factors that plays an important role in neuronal development and the formation of synapses [30]. Neurite outgrowth is the process representing the neuronal differentiation. The activity of ACH on neurite outgrowth formation in high glucose-treated cells was investigated. To create the neurite outgrowth model, SH-SY5Y was cultured in serum starvation, which was low glucose DMEM supplemented with 1% FBS to avoid overgrowth of cells; 10 μM retinoic acid (RA) was added for 24 h [22]. In response to serum starvation and retinoic acid, SH-SY5Y will begin neurite outgrowth formation. Figure 2A shows the comparison of the neurite outgrowth formation between the untreated and RA-treated groups. We found that RA treatment could induce neurite outgrowth, as evidenced by the increased expression of growth-associated protein 43 (GAP-43), a marker of neurite outgrowth, and the increased expression of Teneurin-4, a transmembrane protein that plays a role in the neurogenesis, is highly expressed in the central nervous system, and mediates neurite outgrowth [31,32]. Confirmed by Western blot analysis (Figure 2D–F), the expression of GAP-43 and Teneurin-4 in RA-treated group was increased, and relative GAP-43 and Teneurin-4 expression was 2.94 ± 0.22-fold change and 11.28 ± 0.11-fold change, respectively (^+^ *p* < 0.05 vs. 10 μM RA).

High glucose concentration is a critical factor that could trigger neuronal damage and inhibit neurite outgrowth [1,3]. To examine the effect of high glucose on neurite outgrowth, cells were cultured in high glucose conditions for 48 h. We found that 50 mM of glucose could significantly reduce the neurite outgrowth formation and neurite length when compared to the RA-treated group (^+^ *p* < 0.05) (Figure 2A,C) and GAP-43 expression (1.54 ± 0.04-fold change or 55.15% reduction compared to RA-treated group, Figure 2D,E). Next, we examined the effect of the extracts on the elongation of neuronal processes. Treatment of the cells with 10 or 50 μg/mL ACH was performed. Figure 2A showed that ACH extract could induce the neurite outgrowth in high glucose-treated cells, and Figure 2D–F shows that ACH could also increase the GAP-43 expression in a concentration-dependent manner (2.23 ± 0.09-fold change and 4.01 ± 0.17-fold change, respectively). In addition, Teneurin-4 expression was not altered in both the high glucose-treated group and 10 μg/mL ACH-treated group, while 50 μg/mL ACH could cause a considerable reduction of Teneurin-4 expression (6.61 ± 0.09-fold change or 41.37% reduction compared to RA-treated group). Finally, the number of bearing cells and neurite length are shown in Figure 2B,C, respectively. The number of neurite-bearing cells and neurite length were significantly increased in retinoic acid–treated cells. The number was not altered in both the high glucose-treated group and 10 μg/mL ACH-treated group. However, Figure 2A shows the cell cluster containing more than five cells when treated with ACH (50 μg/mL). These cell clusters were excluded for scoring as neurite-bearing cells; therefore, the number of neurite-bearing cells in the ACH-treated group was decreased (not significant) when compared to the RA-treated group. On the contrary, both concentrations of ACH could significantly increase the length of neurites in a dose-dependent manner compared to the control group, but ACH at the concentration of 50 μg/mL could significantly increase the length of neurites compared to the RA-treated group.

### 3.4. Effects of ACH on Cell Cycle Delay

**Figure 3:**
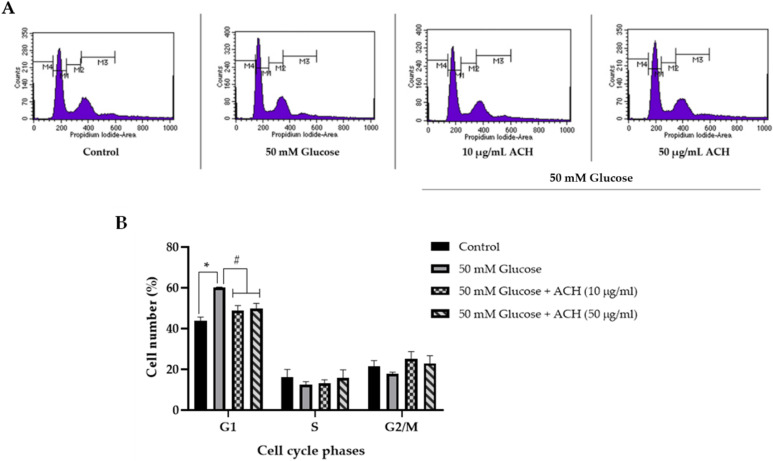

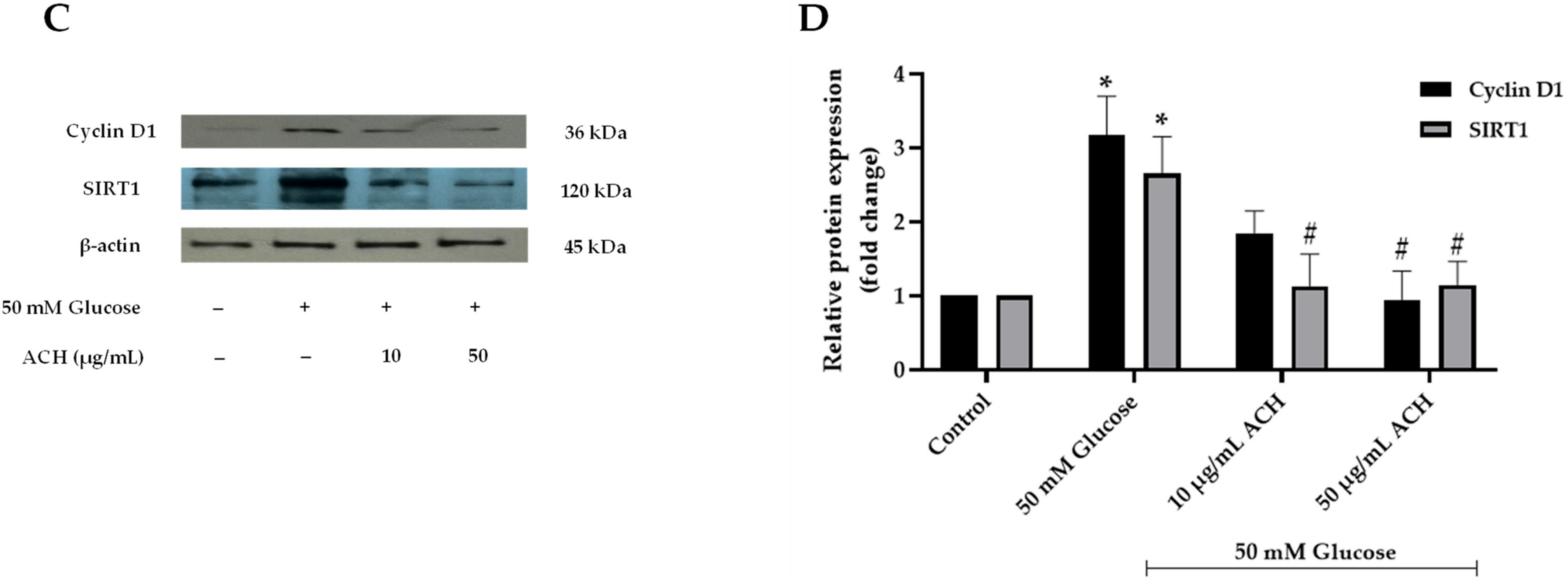
The neuroprotection of ACH on high glucose-induced cell cycle delay. Quantitative determination based on propidium iodide (PI) staining was carried out using a flow cytometer. Cell histogram (**A**) and the percentage of cell numbers (**B**) are shown. Cyclin D1 and sirtuin 1 (SIRT1) are as shown in a representative Western blot (**C**). Normalized values of both cyclin D1 and SIRT1 against β-actin (**D**). The mean ± SEM values of normalized cyclin D1 and SIRT1 expression were obtained from three independent experiments, * *p* < 0.05 vs. control; ^#^ *p* < 0.05 vs. 50 mM glucose.

High glucose level may cause neurotoxicity by interfering with cell cycle progression. A flow cytometer was used for the cell cycle diagram and cell numbers. Figure 3A,B demonstrates the activity of cell cycle distribution and the data generated from the flow cytometer. The percentage of cell numbers in the G1 phase in the high glucose-treated group was significantly higher than the control group (43.79% ± 1.83 for control and 60.03% ± 0.35; for high glucose-treated group), which causes cells to arrest in the resting phase (G0/G1). Post-treatment of ACH extract could attenuate the high glucose effect and normalize cell cycle progression. The percentage of cells in both the 10 and 50 μg/mL ACH treatments was significantly lower than the high glucose treatment (48.68% ± 2.56 and 49.86% ± 2.49, respectively).

We also investigated the expression of two cell cycle-associated proteins, cyclin D1 and SIRT1, using Western blot. Cyclin D1 is the G1 cyclin regulatory partner to control cell cycle progression. As in our previous study, upregulation of cyclin D1 caused cell cycle arrest at G1. Figure 3C,D shows the expression of cyclin D1 was significantly increased when cells were treated with 50 mM glucose alone (* *p* < 0.05 vs. control). The relative cyclin D1 expression was 3.18 ± 1.04 (* *p* < 0.05). When post-treated with ACH, cyclin D1 was significantly reduced in response to treatment with only 50 μg/mL ACH (0.95 ± 0.78; ^#^ *p* < 0.05 vs. high glucose alone).

As shown in Figure 3C,D, the relative SIRT1 expression was significantly increased in response to treatment with high glucose over time (2.66 ± 0.85; * *p* < 0.05 vs. control). Both 10 and 50 μg/mL ACH could normalize cell cycle progression by downregulating SIRT1 expression compared to the 50 mM glucose group (1.13 ± 0.75 and 1.15 ± 0.54; ^#^ *p* < 0.05 vs. high glucose alone, respectively).

### 3.5. ACH Extract Attenuated the High Glucose-Induced Reduction of Body Length and Size and Brood Size

**Figure 4:**
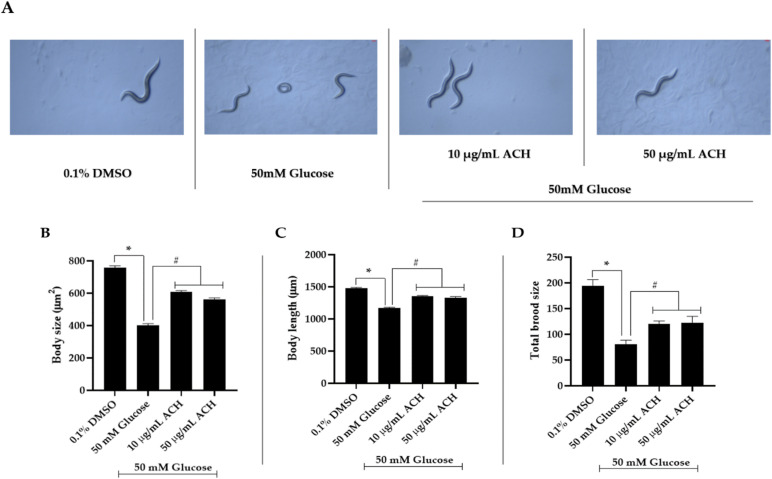
ACH extracts attenuated the high glucose-induced reduction of body length and size and brood size. Bright-field microscope images of *C. elegans* were taken using a 10x objective, and representative images are shown (**A**). The body size and length were measured from at least 20 adult day 1 worms. The number of eggs hatching were counted. The mean ± SEM values of body size (**B**), body length (**C**) and brood size (**D**) are shown, * *p* < 0.05 vs. control; ^#^ *p* < 0.05 vs. 50 mM glucose-fed worms.

At adult day 1 stage, we found differences in both body size and body length following exposure to high glucose levels. Morphological changes were imaged by a 10× objective lens of a bright-field microscope (Figure 4A). The statistical difference between 0.1% DMSO (control group) and the 50 mM glucose-treated group is seen in Figure 4B–D. The body size and length were significantly lower than control group (401.46 ± 10.44 μm^2^ and 1166.89 ± 13.24 μm; * *p* < 0.05). ACH extract co-treatment could work against the effect of high glucose by significantly improving both body size and length compared with high glucose alone. As seen in Figure 4B,C, the body size in 10 and 50 μg/mL co-treatment was 608.13 ± 7.95 μm^2^ and 561.03 ± 10.11 μm^2^; ^#^ *p* < 0.05, respectively. The body length was 1354.38 ± 10.51 μm, ^#^ *p* < 0.05, and 1333.14 ± 13.67 μm, ^#^ *p* < 0.05, in 10 and 50 μg/mL ACH, respectively.

Moreover, we found that high glucose-fed worms showed a significant lower number of eggs laid than the control group (Figure 4D) (80.51 ± 8.10; * *p* < 0.05 vs. control). The total brood size in 10 and 50 μg/mL ACH-fed worms was significantly increased as 119.81 ± 6.04 and 121.90 ± 12.96; ^#^ *p* < 0.05 vs. 50 mM glucose-fed worms, respectively.

### 3.6. ACH Extract against High Glucose-Induced Oxidative Stress in C. elegans

**Figure 5:**
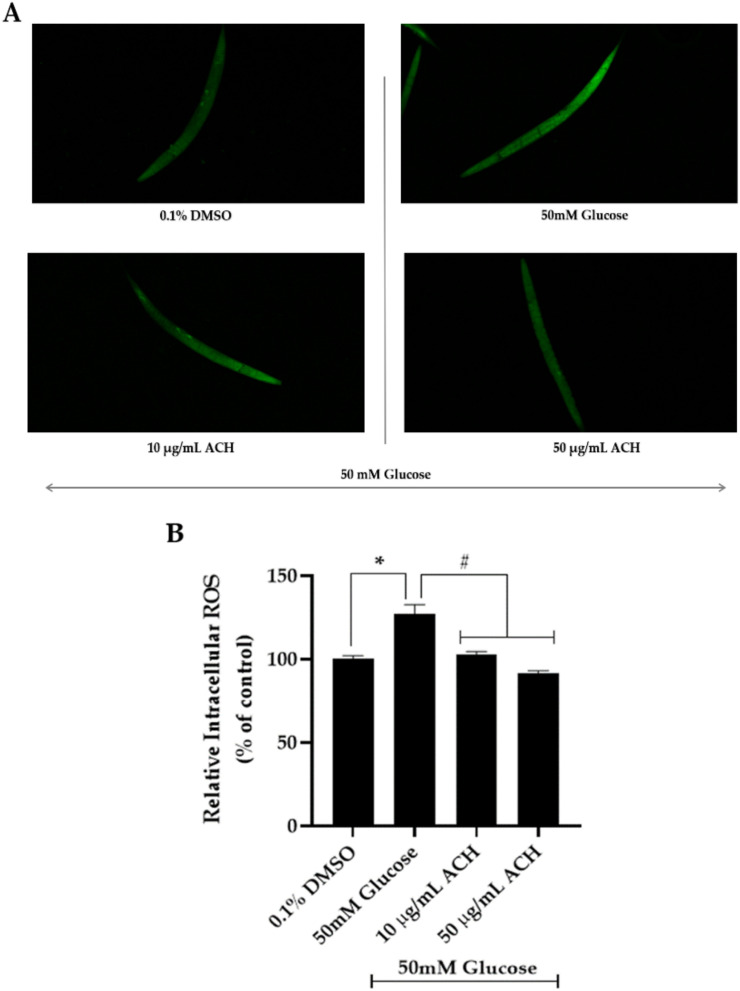
The protective effect of ACH extracts on high glucose-induced oxidative stress in *C. elegans*. Intracellular ROS accumulation was performed using H2DCFDA assay and imaged by a confocal microscope (**A**). Relative intracellular ROS accumulation level is shown in (**B**). Data are presented as the means ± SEM, * *p* < 0.05 vs. control; ^#^ *p* < 0.05 vs. high glucose-fed worms.

The effect of ACH extracts on intracellular ROS production was investigated. Treatment of *C. elegans* with high glucose alone induced intracellular ROS production (127.00 ± 5.71%). However, cultured with both concentrations of ACH, it could attenuate the accumulation of intracellular ROS in a dose-dependent manner (Figure 5A,B) compared with the high glucose-fed worms (102.97 ± 1.62% and 91.46 ± 1.59% in 10 and 50 μg/mL ACH-fed worms, respectively). These results confirmed that ACH extract protects against high glucose-induced healthspan reduction by suppressing ROS formation.

### 3.7. ACH Extracts Extend Lifespan in High Glucose-Fed Worms

**Figure 6:**
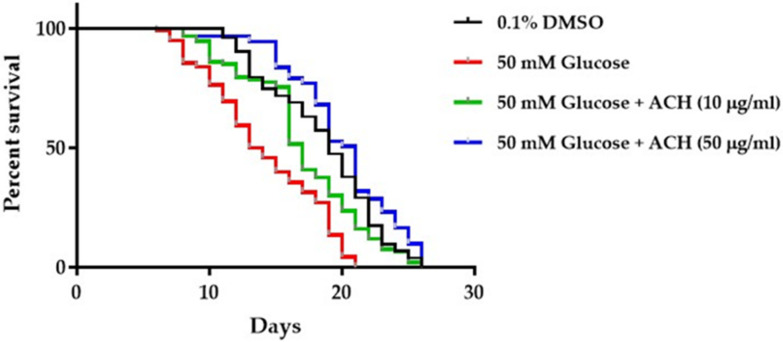
The effect of high glucose and ACH crude extracts on *C. elegans* lifespan.

To investigate the effect of ACH extracts on high glucose-induced lifespan reduction, N2 synchronized L1 larvae were cultured in high glucose alone or high glucose combined with ACH extracts. The results indicated that the mean lifespan of high glucose-fed worms was shorter than 0.1% DMSO-treated worms (approximate 31%). Moreover, ACH could increase the mean lifespan of high glucose-fed worms in a concentration-dependent manner (20.13% and 40.75% compared to mean lifespan of control group). All results are shown in Figure 6 and Table 2.

### 3.8. ACH Extracts Mediated Extension of Lifespan and Healthspan in High Glucose-Fed Worms through DAF-16/FoxO and aqp-1

**Figure 7:**
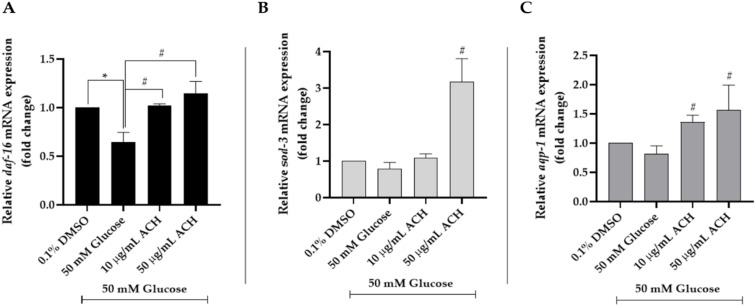
The mRNA expression: (**A**) *daf-16*, (**B**) *sod-3*, and (**C**) *aqp-1* genes of ACH treatment on high glucose-fed worms. All data were presented as the mean ± SEM), * *p* < 0.05 vs. control; ^#^ *p* < 0.05 vs. high glucose-fed worms.

To investigate the effect of ACH extract on mRNA expression, *C. elegans* was cultured and treated as explained earlier. Compared to the vehicle control, we found that high glucose (50 mM) could decrease mRNA expression of *daf-16* (0.65 ± 0.1-fold; * *p* < 0.05), *sod-3*, and *aqp-1*. All mRNA expression could be increased after ACH treatment. The highest change was found in the *sod-3* mRNA expression in 50 μg/mL ACH-fed worms (^#^ *p* < 0.05 vs. high glucose-fed worms). All ACH concentrations significantly increased both *daf-16* and *aqp-1* expression in the concentration-dependent manner. All results are shown in Figure 7.

### 3.9. Phytochemical Constituents of ACH

The GC-MS/MS chromatogram of ACH extract showed 10 major peaks, and the components corresponding to the peaks were determined as follows: 24-methylenecycloartan-3-one (14.17%), squalene (13.55%), D:A-friedooleanan-3-ol, (3.α.)–(10.21%), tritriacontane (9.38%), vitamin E (8.94%), β-amyrin (5.17%), 9,19-cyclolanostan-3-ol,24,24-epoxymethano-,acetate (3.74%), lupenone (lup-20(29)-en-3-one) (3.31%), hentriacontane (2.68%), olean-12-en-3-one (2.23%), and other compounds showed low of percentage of peak area (Table 3).

### 3.10. The Ability of ACH-Derived Phytochemical Constituents as Inhibitors of IGFR Using an In Silico Approach

Next, we investigated the competence of ACH on IGFR inhibition and identified which phytochemicals were more efficient than the binding of each positive control. At least 27 phytochemicals obtained from GC-MS/MS were analyzed by molecular docking analysis.

In the current experiment, EGCG and resveratrol were used as the positive control in the molecular docking study. EGCG has been previously reported as an inhibitor of the activity of IGFR [33]. In addition, resveratrol exerted suppression on IGF-1 [34,35,36].

In Table 4, the binding energy of −9.88, −6.54, and −6.57 kcal/mol was exerted by the original ligand, EGCG, and resveratrol, respectively.

Based on the docking results in Table 4, five phytochemicals showed outstanding inhibition against IGFR, with higher binding energy (higher than −9 kcal/mol) compared to the others; noticeably, their binding energy was also higher than both positive controls. The five phytochemical constituents included olean-12-en-3-one, lupenone, stigmasterol, α-amyrin, and β-amyrin. Interestingly, 2D diagrams of the five phytochemicals, original ligand, and both positive controls are shown in Figure S1 in the Supplementary Materials for IGFR.

For the method validation, 2-[4-[4-[[(6Z)-5-chloranyl-6-pyrazolo [1,5-a]pyridin-3-ylidene-1H-pyrimidin-2-yl]amino]-3,5-dimethyl-pyrazol-1-yl]piperidin-1-yl]-N,N-dimethyl-ethanamide, a reported inhibitor of the IGFR (5FXS) crystal structure, was removed and re-docked into the original active cavity of the protein. The results showed that the original inhibitor was capably re-docked into the similar location and orientation of the native crystal structure with RMSD 2.48 Å (less than 2.5 Å is considered a near-native solution, and 2–3 Å is acceptable for docking [doi.org/10.1002/prot.24214, doi.org/10.3390/molecules23051038]). Moreover, the predicted binding energy was −9.88 kcal/mol, demonstrating the acceptable reproducibility of analysis. Protein–ligand interactions showed that re-docking conformation of the original interacted with key amino acids found in the co-crystallized structure. The re-docking showed that the original ligand formed a hydrogen bond with two amino acids found in the co-crystallized complex: GLU1080 and MET1082. In addition, it shared amino acid interaction with five out of six amino acids: LEU1005, VAL1013, ALA1031, LYS1033 and LEU1081, by hydrophobic bonding. Therefore, these results indicate that the protocol used in this study is reliable and could be applied for further predictions.

### 3.11. Isolation and Chemical Characterization of Active Compounds in ACH Extract

In order for active compounds to be derived from ACH to investigate the protective effect against high glucose-induced toxicity, ACH crude extract (1.2 g) was isolated by column chromatography using the condition hexane:ethyl acetate (EtOAc) (90:10). As a result, the eluates were examined by TLC, and seven combined fractions (ACH1-ACH7) were obtained.

**Figure 8:**
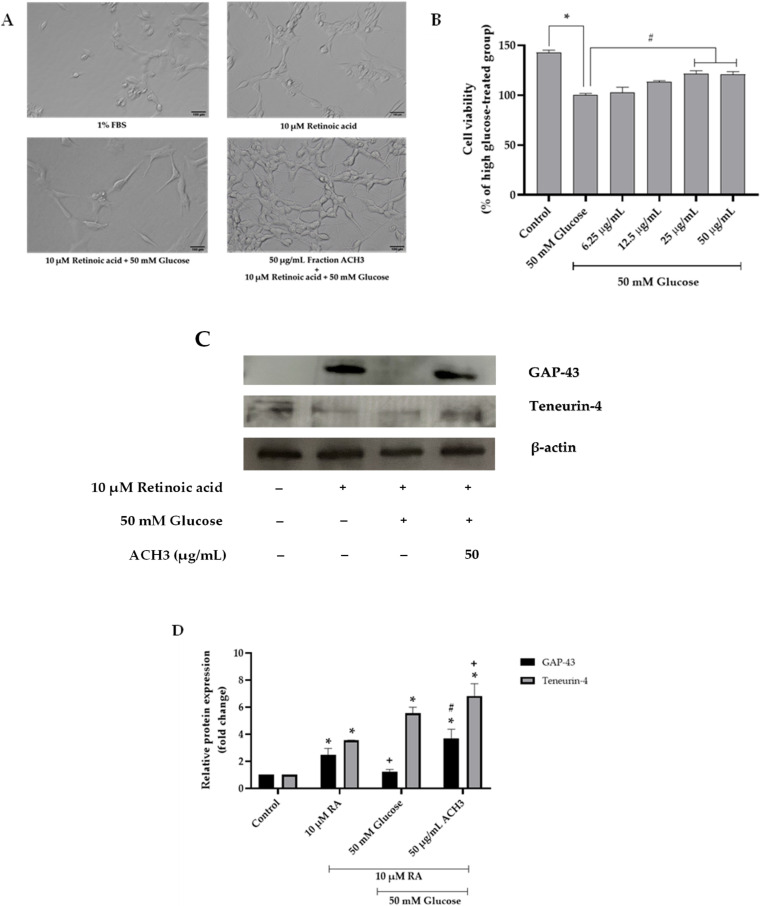
The effect of fraction ACH3 on neurite outgrowth (**A**). MTT assay was performed to clarify the effect of fraction ACH3 on cell viability (**B**). GAP-43 and Teneurin4 expression shown in a representative Western blot (**C**). Normalized values of both GAP-43 and Teneurin-4 against β-actin (**D**). All data are presented as the mean ± SEM, * *p* < 0.05 vs. control; ^+^ *p* < 0.05 vs. 10 μM RA; ^#^ *p* < 0.05 vs. high glucose-treated cells.

We further investigated the effect of fraction ACH on high glucose-induced neurotoxicity. Neurite outgrowth-promoting activities of fraction ACH were evaluated in SH-SY5Y cells. We found that only ACH3 could exert remarkable neuroprotection by attenuating high glucose-induced neurite outgrowth inhibition (Figure 8A). The protection of this fraction on cell viability in high glucose concentrations was determined. Only ACH3 at the concentrations of 25 and 50 μg/mL protected SH-SY5Y cell viability from high glucose-induced damage (Figure 8B). In addition, Western blot results (Figure 8C,D) showed that ACH3 (50 μg/mL) could attenuate high glucose-inhibited neurite outgrowth through the induction of GAP-43 and Teneurin-4 expression. Together, we propose that this shows that fraction ACH3 is a probable active fraction.

Next, fraction ACH3 was subjected to column chromatography on silica gel using hexane:EtOAc (90:10). Compound **1** (10.1 mg) and **2** (50.2 mg) were afforded from ACH3. Their structures were further elucidated using ^1^H-NMR.

The results indicated that a mixture of Compounds **1** and **2** was stigmastrol and β-sitosterol. The structure of these compounds is shown in Figure S2 in the Supplementary Material. The spectrum of these compounds obtained from ^1^H-NMR is shown in Figure S3 in the Supplementary Material. Chemical information on these isolated compounds is as follows.

### 3.12. Compound ***1*** and ***2*** (A Mixture of Stigmastrol and β-Sitosterol)

White powder; ^1^H-NMR (CDCl_3_, 400 MHz): 0.65 (*s*, 3H), 0.80 (*d*, 3H, *J* = 6.4), 0.82 (*d*, 3H, *J* = 6.4), 0.84 (*t*, 3H, *J* = 7.2), 0.98 (*d*, 3H, *J* = 6.5), 1.02 (*s*, 3H), 3.50 (*tdd*, 1H, *J* = 4.5, 4.2, 3.8), 4.98 (*m*, 1H), 5.14 (*m*, 1H), 5.32 (*t*, 1H, *J* = 6.4).

Finally, the presence of β-sitosterol and stigmasterol was evaluated by using reverse-phase high-performance liquid chromatography (RP–HPLC). The HPLC fingerprints are shown in Figure S4 in the Supplementary Material. The amount of β-sitosterol and stigmasterol in ACH extract was 54.11 ± 0.5 ppm and 22.47 ± 0.42 ppm, respectively.

## 4. Discussion

*Aquilaria crassna* (AC) is a fragrant and medicinal plant used in many traditional medicines. We studied the protective effect of AC on high glucose-induced neurotoxicity and aging. To create a high glucose-induced neurotoxicity model, 50 mM glucose was selected, as a previous report [4] showed that *C. elegans* culturing under high glucose condition (50 mM) resulted in a glucose concentration in whole-body extracts of approximate 15 mM. At 10–15 mM glucose, it resembles the glucose concentrations in diabetic patients under poor glucose control. Furthermore, 50 mM glucose could induce nitric oxide production, protein kinase C activation, myo-inositol metabolism alteration, and defective tissue perfusion in high glucose-treated SH-SY5Y cells. The above-mentioned process involved the pathogenesis of diabetic complications, including neuropathy [37].

First, ACH supported the neuroprotective effect on high glucose-inhibited neurite outgrowth. The neuronal polarization is a crucial step that consists of two importantly different processes via a long axon and several short dendrites. These processes are associated with brain functions such as memory, learning, and emotion [38]. The profound outgrowth of axons was used as evidence of neuronal polarization [39]. In addition, GAP-43 and Teneurin-4 proteins were used as the important markers of neuronal polarization in this study. During the early stage of neurite outgrowth, filopodia are formed through the protrusion of small cell membranes. Teneurin-4 is a transmembrane protein and a positive regulator that functions in filopodia-like protrusion formation and neurite outgrowth [31]. Additionally, GAP-43 is another positive regulator during neurite outgrowth development [40]. The ACH extract on neurite outgrowth results (Figure 2) indicated that ACH could attenuate high glucose-inhibited neurite outgrowth. Interestingly (Figure 2B,C), we found that high glucose could only reduce GAP-43 expression; it did not alter Teneurin-4 expression. This could be described as high glucose reducing the neurite outgrowth development but not affecting the filopodia-like formation. The level of 50 μg/mL ACH could cause the considerable reduction of Teneurin-4 expression; this concentration of ACH could induce neurite outgrowth development but did not increase the number of filopodia-like formations. These data were in accordance with the number of neurite-bearing cells (or filopodia-like formations) (Figure 2D) and the neurite length (Figure 2E).

For cell cycle results (Figure 3A,B), high glucose causes cell cycle arrest at the G1 phase, and ACH normalized the cell cycle progress in high glucose-treated cells by significantly decreasing the percentage of cell number at the G1 phase. To confirm the effect of ACH on the cell cycle, the expression of proteins cyclin D1 and SIRT1 was detected by Western blot. SIRT1, also known as NAD-dependent deacetylase sirtuin-1, plays an important role in normalizing cell cycle progression, inhibiting cell senescence, and extending the lifespan of organisms [41,42,43]. Our previous study [20] confirmed the function of SIRT1 on high glucose-induced cell cycle delay and in response to glucose starvation. When the glucose level in cells was low, SIRT1 expression in the nucleus was activated and upregulated by the induction of phosphorylated GAPDH. In contrast, Figure 3C,D showed the different results. Western blot and normalized SIRT1 values revealed that treating cells with 50 mM glucose for 48 h could significantly increase SIRT1 expression. This process shows that the other important factor in addition to the glucose level was the time-dependent manner. SIRT1 expression was transiently downregulated by high glucose treatment. Then, cells were treated with high glucose over time. SIRT1 expression level gradually increased from 24 h to 48 h [44]. Our result (Figure 1A) indicated that high glucose could cause cellular oxidative stress. SIRT1 is triggered and redistributed in the mechanism responsible for maintaining cell homeostasis under oxidative stress [45,46]. Therefore, it could be described as why the level of SIRT1 expression was significantly increased in high glucose-treated cells.

*Caenorhabditis elegans* or *C. elegans* can be widely used as a model for the study of the molecular target and mechanisms affected by the pathological glucose concentrations. *C. elegans* has its short lifespan, and easily modifiable genome, and a simple glucose receptor system [47]. To examine the effect of high glucose concentration on the development of *C. elegans*, L1 larvae stage worms were cultured in NGM agar supplemented with *E. coli* OP50 and 50 mM glucose. This glucose concentration resulted in the intracellular glucose concentration of 14 to 15 mM and was sufficient to achieve significant effects on the development, offspring, and lifespan of *C. elegans*.

It has been documented that long-lasting high glucose conditions result in the substantial accumulation of the modification of mitochondrial proteins and a steady increase in ROS formation [48]. ROS plays an important role in the reduction of lifespan and healthspan in *C. elegans*. It well known that under stress conditions, a large amount of ROS will be released. ROS was associated with implications for pathogenesis of many diseases; in addition, it is one of the major causes of aging. Our previous research publications indicated that a certain concentration of antioxidants will contribute to the longevity of *C. elegans* [49].

To explain the mechanisms by which high glucose induced the reduction of lifespan and ACH extracts attenuated the toxicity of high glucose, we combined our results and found that high glucose caused lifespan reduction by interfering with worm development (such as body length and size), reducing in the number of progeny or eggs, and increasing the accumulation of intracellular ROS.

High glucose concentration disrupted the activation of genes associated with the protective mechanisms and caused shortened lifespan and reduced healthspan of *C. elegans*. Insulin and its close homolog insulin-like growth factor 1 (IGF-1) could bind to the tyrisine-kinase receptor, resulting in the inhibition of the forkhead boxO (FoxO) transcription factor. This transcription factor plays a vital role in many cellular processes, such as stress resistance, energy metabolism, apoptosis, cell cycle arrest, and organism longevity [50,51]. The FoxO family member *daf-16* and its transcription target gene, including *sod-3,* are important key factors contributing to the mediation of lifespan extension, metabolism, and stress response [27,52]. In addition, *daf-16* could act as a cumulative way for other genes to have an influence on lifespan [53]. As recently shown, the lifespan of *C. elegans* was significantly shortened by a high glucose-enriched diet through inhibiting the transcription factor *daf-16*. In addition, the role in response to glucose-induced lifespan shortening was associated with the reduction of the activity of the downstream aquaporin gene *aqp-1* [54]. This resulted in the *aqp-1* gene being noteworthy. The authors found a large impact on lifespan related to the loss of *aqp-1*. The *aqp-1* is a regulatory gene required for *daf-16*-regulated gene expression, including normalization of *sod-3* expression. Therefore, *aqp-1* might be a feedback regulator of insulin/IGF-1 signaling.

These reasons are consistent with our current results. ACH extract could attenuate the effect of glucose on shortening *C. elegans* lifespan through improving the mRNA expression of the glucose-responsive *daf-16* target gene, *sod-3*, and *aqp-1*. Collectively, ACH extract might be an interesting neuroprotectant and anti-aging agent. As shown in the above-mentioned experimental results, high glucose shortens the lifespan and reduces the healthspan of *C. elegans*, and ACH extract can attenuate the influence of high glucose. Conserved from *C. elegans* to mammals, many studies have indicated that glucose stimulated insulin secretion in *C. elegans* and is related to insulin-like growth factor 1 receptor (IGFR) and insulin/IGF-1 signaling [50,54,55]. Reducing the GFR and IGF-1 signaling pathway slows the aging process, doubles lifespan, and improves healthspan [56,57].

Next, various phytochemicals contained in ACH were analyzed and identified by GC-MS/MS. Moreover, the binding affinity of the phytochemicals of ACH against IGFR was analyzed by in silico analysis. The results revealed that five phytochemicals of olean-12-en-3-one, lupenone, stigmasterol, α-amyrin, and β-amyrin were more efficient than the binding of each positive control (EGCG or resveratrol).

Analyzed and confirmed by NMR and RP-HPLC, fraction ACH3 consisted of both active compounds: β-sitosterol and stigmasterol. Phytosterol is a class of natural products found in food, cosmetics, and medicinal plants such as AC. Along with over 200 types, stigmasterol and β-sitosterol are the most extensive in many plants [58]. There were many studies indicating that phytosterol might play an important role in the prevention of neurodegenerative disorders. Stigmasterol exerted neuroprotection against oxidative stress-induced neurodegeneration by upregulating FoxO, catalase, anti-apoptotic protein B-cell lymphoma 2 (Bcl-2), and SIRT1 expression in neurons [59]. Moreover, stigmasterol possesses neuroprotective activities against oxidative stress-indued ischemic injury and autophagy [60]. Stigmasterol could also decrease the activity of beta-secretase enzyme (BACE1) and the β-secretase cleavage of amyloid precursor protein (APP) [61]. On the other hand, β-sitosterol could inhibit neuroinflammation in neurodegenerative diseases through repressing pro-inflammatory markers, including interleukin-6, inducible nitric oxide, cyclooxygenase-2, and the phosphorylation of nuclear factor kappa B [62]. β-sitosterol prevented mitochondria dysfunction by increasing the mitochondrial ATP concentrations and mitochondrial potential [63]. Moreover, β-sitosterol could increase antioxidant enzymes through activating the estrogen receptor/PI3-kinase pathway, exerting anti-acetyl choline esterase (AChE) and anti-butyl choline esterase (BChE) inhibitory potentials in both in in vitro and in vivo models, and also playing a role as a free radical scavenger by regulating the glutathione level [64]. In our previous study, stigmasterol was a probable active component of *Momordica charantia* extract for the prevention for Polycyclic aromatic hydrocarbons (PAHs)-induced neurotoxicity [19]. β-sitosterol and stigmasterol have also been reported to possess the strongest neurite outgrowth-promoting activities of four sterol compounds in neuronal cell culture [65]. In accordance with this study, fraction ACH3 (50 μg/mL) exerted the neuroprotection of high glucose-inhibited neurite outgrowth. Therefore, β-sitosterol and stigmasterol are potentially interesting neuroprotectants derived from the ACH extract.

## 5. Conclusions

In summary, the AC leaf is a beneficial plant with rich bioactive compounds. ACH represents neuroprotection from high glucose-induced neuronal cell damage, including the induction of GAP-43/Teneurin-4-mediated neurite outgrowth and cell cycle normalization via the cyclin D1/SIRT1 signaling pathway. ACH also exerts oxidative resistance properties, healthspan improvement, and lifespan extension via the *daf-16*/FoxO and *aqp-1* pathway. At least 27 phytochemical compounds were identified by GC-MS/MS. Moreover, molecular docking analysis and NMR reveal that β-sitosterol and stigmasterol are bioactive phytochemicals in ACH. Collectively, ACH could be developed as an agent for the protection of high glucose-induced neurotoxicity and aging.

## Figures and Tables

**Table 1:** The sequence of RNA primers.

Primer	Forward 5′-3′	Reverse 5′-3′
*daf-16*	TTTCCGTCCCCGAACTCAA	ATTCGCCAACCCATGATGG
*sod-3*	TTCGAAAGGGAATCTAAAAGAAG	GCCAAGTTGGTCCAGAAGATAG
*aqp-1*	TTTTGGCAAGGAACCTCATC	GCTGTTGCCATAACTGCAAA
*act-1*	AGACAATGGATCCGGAATGT	CATCCCAGTTGGTGACGATA

**Table 2:** Results and statistical analyses of lifespan of *C. elegans* treated with high glucose and ACH.

Groups	Mean Lifespan		*p*-Value (vs. Control)	*p*-Value (vs. 50 mM Glucose)	Number of Worms
	Day ± SEM	% Increase (vs. 50 mM Glucose)			
0.1% DMSO	18.55 ± 0.42	31.00	-	0.0001	102
50 mM Glucose	14.16 ± 0.40	-	0.0001	-	118
50 mM Glucose + 10 μg/mL ACH	17.01 ± 0.47	20.13	0.0379	0.0001	93
50 mM Glucose + 50 μg/mL ACH	19.93 ± 0.43	40.75	0.0192	0.0001	91

**Table 3:** Proposed phytochemical constituents in ACH extract compared with the National Institute of Standards and Technology (NIST) database.

Compound	RT	Area (%)	MF	MW
tetradecane	22.867	0.15	C_14_H_30_	198
hexadecane	30.730	0.12	C_16_H_34_	226
phytol	39.231	0.3	C_20_H_40_O	296
n-hexadecanoic acid	43.3	0.9	C_16_H_32_O_2_	256
oleic acid	48.638	0.57	C_18_H_34_O_2_	282
oleamide (9-Octadecenamide, (Z)-)	54.799	0.97	C_18_H_35_NO	281
squalene	66.506	13.55	C_30_H_50_	410
nonacosane	68.036	0.49	C_29_H_60_	408
9,19-cyclolanost-24-en-3-ol, acetate, (3.β.)	68.794	0.38	C_32_H_52_O_2_	468
2,2,4-trimethyl-3-(3,8,12,16-tetramethyl-heptadeca-3,7,11,15-tetraenyl)-cyclohexanol	69.037	0.4	C_30_H_52_O	428
ϒ-tocopherol	71.233	0.69	C_28_H_48_O_2_	416
hentriacontane	72.371	2.68	C_31_H_64_	436
vitamin E	73	8.94	C_29_H_50_O_2_	430
stigmasterol	75.305	1.17	C_29_H_48_O	412
D-friedoolean-14-en-3-one	76.122	1.25	C_30_H_48_O	424
tritriacontane	76.463	9.38	C_33_H_68_	464
β-amyrin	76.547	5.17	C_30_H_50_O	426
olean-12-en-3-one	77.411	2.23	C_30_H_48_O	424
lupenone (lup-20(29)-en-3-one)	77.498	3.31	C_30_H_48_O	424
α-amyrin	77.878	1.94	C_30_H_50_O	426
lup-20(29)-en-3-ol, acetate, (3.β.)	78.077	0.93	C_32_H_52_O_2_	468
D:A-friedooleanan-7-one, 3-hydroxy	78.41	1.19	C_30_H_50_O_2_	442
ursa-9(11),12-dien-3-ol	78.65	1.16	C_30_H_48_O	424
9,19-cyclolanostan-3-ol,24,24-epoxymethano, acetate	78.796	3.74	C_33_H_54_O_3_	498
betulin	79.05	1.00	C_31_H_52_O	440
friedelan-3-one	79.473	0.42	C_30_H_50_O	426
D:A-friedooleanan-3-ol, (3.α.)	80.004	10.21	C_30_H_52_O	428
pentatriacontane	80.197	1.42	C_35_H_72_	492
24-methylenecycloartan-3-one	80.474	14.17	C_31_H_50_O	438

RT: retention time; MF: molecular formula; MW: molecular weight.

**Table 4:** Docking results of the compounds with IGFR.

No.	Compound	MW	Binding Energy (kcal/mol)	Inhibition Constant	Amino Acid Interaction		
					Hydrogen Bond	Hydrophobic Bond	Electrostatic Bond
	EGCG (positive control)	458.4	−6.54	16.18 μM	ASP1086 SER1089 GLU1015	LEU1005 (2) ARG1084 (2) GLY1085	
	Resveratrol (positive control)	228.24	−6.57	15.39 μM	MET1082 MET1082 GLU1080 GLN1007	VAL1013 ALA1031 MET1156	
1	24-Methylenecycloartan-3-one	438.7	−5.13	172.22 μM	GLY1085	LEU1005 VAL1013 (2) ALA1031 LYS1033 MET1079 LEU1081 (2) MET1082 MET1142 MET1156 (2)	
2	Squalene	410.7	−6.37	21.57 μM		ARG1003 LEU1005 (3) VAL1013 ALA1031 (2) LYS1033 VAL1063 MET1079 LEU1081 (2) MET1142 (2) MET1156 (2)	
3	D:A-Friedooleanan-3-ol, (3.alpha.)-	428.7	−5.90	47.43 μM	GLU1080	VAL1013 ALA1031 ALA1031 MET1142 VAL1063 MET1079 MET1142 VAL1013 LYS1033 MET1079 VAL1013 MET1156	
4	Friedelan-3-one	426.7	−7.88	1.66 μM		VAL1013 (3) ALA1031 (3) LYS1033 (2) MET1079 MET1142 MET1156	
5	Stigmasterol	412.7	−9.32	146.8 nM	ARG1003	LEU1005 (3) VAL1013 (2) LEU1081 MET1142 (2)	
6	Tritriacontane	464.9	−3.51	2.69 mM		LEU1005 (3) ALA1031 (2) LYS1033 MET1079 MET1142 (4) MET1156 (2) ILE1160	
7	Vitamin E	430.7	−7.92	1.56 μM	GLY1085 ASP1086 SER1089	VAL1013 (3) ALA1031 (2) LYS1033 MET1079 MET1082 MET1142 (2) MET1156 ILE1160	**ASP1086**
8	D-Friedoolean-14-en-3-one	424.7	−8.88	355.27 nM		LEU1005 (4) VAL1013 ALA1031 LEU1081 (2) MET1142	
9	9,19-Cyclolanostan-3-ol,24,24-epoxymethano-, acetate	498.8	−7.30	4.49 μM	SER1089 GLY1085 MET1082	LEU1005 VAL1013 (2) ALA1031 (2) LYS1033 MET1142 (2) MET1156 (2) ILE1160 MET1079 (2) LEU1081	
10	D:A-Friedooleanan-7-one, 3-hydroxy-	442.7	−5.37	116.19 μM	GLU1080	LEU1005 VAL1013 ALA1031 MET1079 MET1082 MET1142 (2)	
11	Beta-amyrin	426.7	−9.02	245.77 nM	THR1083	LEU1005 VAL1013 (4) ALA1031 LYS1033 (3) MET1142 (2) MET1156 (2)	
12	Olean-12-en-3-one	424.7	−9.77	68.61 nM		LEU1005 VAL1013 (3) ALA1031 (3) LYS1033 (2) MET1079 (2) MET1142 (3) MET1156	
13	Lup-20(29)-en-3-ol, acetate, (3.beta.)-	468.8	−7.36	4.02 μM		LEU1005 VAL1013 ALA1031 (3) VAL1063 MET1079 LEU1081 MET1142 (3)	
14	Lupenone (Lup-20(29)-en-3-one)	424.7	−9.56	97.57 nM	SER1089	LEU1005 VAL1013 ALA1031 (2) LYS1033 (2) MET1142 (3) MET1156 (2) VAL1063 MET1079 LEU1081 MET1082	
15	Gamma-Tocopherol	416.7	−7.75	2.08 μM		LEU1005 (3) VAL1013 (2) LYS1033 MET1079 LEU1081 MET1142 MET1156 (2)	
16	Hentriacontane	436.8	−3.39	3.25 mM		LEU1005 (2) VAL1013 (2) ALA1031 (2) LYS1033 LEU1081 MET1142 (2) MET1156 ILE1160 (2) TYR1161	
17	Oleamide (9-Octadecenamide, (Z)-)	281.5	−4.51	490.34 μM	GLU1080	LEU1005 (2) VAL1013 (3) ALA1031 LYS1033 (2) MET1079 MET1156	
18	Alpha-amyrin	426.7	−9.21	178.76 nM		LEU1005 VAL1013 (2) ALA1031 (3) LYS1033 MET1079 MET1142 (2) MET1156	
19	Pentatriacontane	492.9	−1.59	68.88 mM		LEU1005 (5) VAL1013 ALA1031 ARG1084 MET1142	
20	Ursa-9(11),12-dien-3-ol	424.7	−8.53	554.63 nM		VAL1013 (2) ALA1031 LYS1033 ILE1160 VAL1063 LEU1081 MET1082 (2) MET1142 (2) MET1156	
21	Betulin	442.7	−8.86	322.86 nM		LEU1005 (2) ALA1031 (3) VAL1063 MET1079 MET1082 MET1142 (4) MET1156	
22	n-Hexadecanoic acid	256.42	−3.76	1.75 mM	MET1082 GLU1080	LEU1005 VAL1013 (3) ALA1031 LYS1033 (2) MET1079 MET1156 (2)	
23	Oleic acid	282.5	−4.43	564.46 μM	SER1089 ASP1086	VAL1013 (2) ALA1031 (2) LYS1033 MET1142 (2) MET1156 (2)	
24	Nonacosane	408.8	−3.85	1.52 mM		LEU1005 VAL1013 (3) ALA1031 (2) LYS1033 (2) VAL1063 MET1079 (2) MET1142 MET1156 (2)	
25	2,2,4-Trimethyl-3-(3,8,12,16-tetramethyl-heptadeca-3,7,11,15-tetraenyl)-cyclohexanol	428.7	−6.75	11.23 μM	SER1089	LEU1005 (2) VAL1013 (2) ALA1031 (3) LYS1033 MET1079 MET1142 (2) MET1156 (2)	
26	9,19-Cyclolanost-24-en-3-ol, acetate, (3.beta.)-	468.8	−5.29	132.45 μM		LEU1005 (3) ALA1031 (2) MET1079 ARG1084 TYR1090 MET1142 (2)	
27	Phytol	296.5	−5.17	161.26 μM	ASP1086	LEU1005 (2) VAL1013 (3) ALA1031 (2) LYS1033 (2) LEU1081 MET1082 MET1142 (2) MET1156	

## Data Availability

Not applicable.

## Supplementary Materials

The following supporting information can be downloaded at: https://www.mdpi.com/article/10.3390/nu14173668/s1, Table S1: The results of method validation between co-crystalized ligand at the original active site of IGFR (PDB ID: 5FXS); Figure S1: The results of the molecular docking study of IGFR were represented by 2D diagram of phytochemical-receptor interactions; Figure S2: Structure of compound **1** (stigmasterol) and compound **2** (β-sitosterol) from fraction ACH3; Figure S3: ^1^H-NMR spectrum of a mixture of β-sitosterol and stigmastrol (compound **1** and **2**) in CDCl_3_; Figure S4: Reverse-phase HPLC analysis of standard β-sitosterol, stigmasterol and the extract of ACH showing the presence of both β-sitosterol and stigmasterol.

## Abbreviations

AC	*Aquilaria crassna*
ACH	AC hexane extract
GAP-43	Growth-associated protein 43
SIRT1	Surtuin-1
GAPDH	Glyceraldehyde-3-phosphate dehydrogenase
DAF-16/FoxO	Forkhead box protein O
*sod-3*	Superoxide dismutase-3
*aqp-1*	Aquaporin-1
ROS	Reactive oxygen species
H_2_DCFDA	2,7-Dichlorofluorescein diacetate
ABTS	2,2-Azino-bis(3-ethylbenzothiazoline-6-sulfonic acid)
DPPH	Diammonium salt, 2,2-diphenyl-1-picrylhydrazyl
DMSO	Dimethyl sulfoxide
GC-MS/MS	Gas chromatography mass spectrometry/mass spectrometry
NMR	Nuclear magnetic resonance
RP-HPLC	Reverse-phase high-performance liquid chromatography
TLC	Thin layer chromatography
VCEAC	Vitamin C equivalent antioxidant capacity
IGFR	Insulin-like growth factor 1 receptor
EGCG	Epigallocatechin gallate
