# Supplementary material for: Aquilaria crassna Leaf Extract Ameliorates Glucose-Induced Neurotoxicity In Vitro and Improves Lifespan in Caenorhabditis elegans
Source: Nutrients. 2022 Sep 5;14(17):3668. doi: 10.3390/nu14173668 (PMC9460374; doi:10.3390/nu14173668)
Supplement: Supplementary file 1 [file nutrients-14-03668-s001.zip › nutrients-1840757-supplementary.pdf]

Table S1. The results of method validation between co-crystalized ligand at the original active site of IGFR (PDB ID: 5FXS)

Ligand	RMSD (Å)	Binding energy (kcal/mol)	Amino acid interaction		
			Hydrogen bond	Hydrophobic bond	Electrostatic bond
**2-[4-[4-[[(6Z)-5-chloranyl-6-pyrazolo[1,5-a]pyridin-3-ylidene-1H-pyrimidin-2-yl]amino]-3,5-dimethyl-pyrazol-1-yl]piperidin-1-yl]-N,N-dimethyl-ethanamide (crystal structure)**	Not determined	Not determined	GLU1080<br>MET1082	LEU1005 (2)<br>VAL1013<br>ALA1031<br>LYS1033<br>MET1142<br>LEU1081	-
**2-[4-[4-[[(6Z)-5-chloranyl-6-pyrazolo[1,5-a]pyridin-3-ylidene-1H-pyrimidin-2-yl]amino]-3,5-dimethyl-pyrazol-1-yl]piperidin-1-yl]-N,N-dimethyl-ethanamide (re-docking)**	2.48	-9.88	GLU1080<br>MET1082<br>ASP1086 (2)	LEU1005 (3)<br>VAL1013 (3)<br>ALA1031 (2)<br>LYS1033 (2)<br>MET1079<br>LEU1081	-

Original ligand

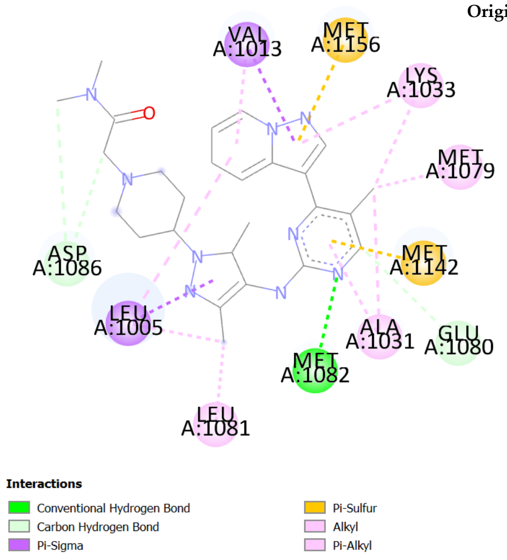

EGCG

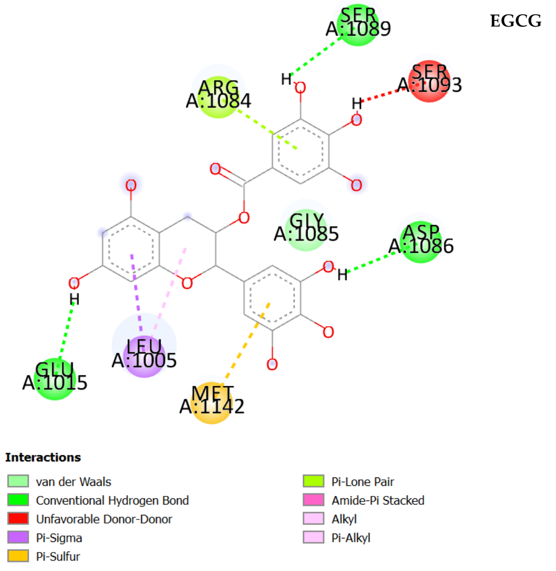

Resveratrol

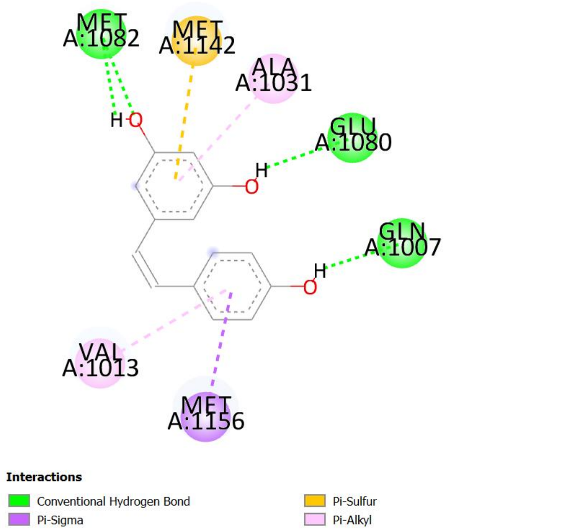

Olean-12-en-3-one

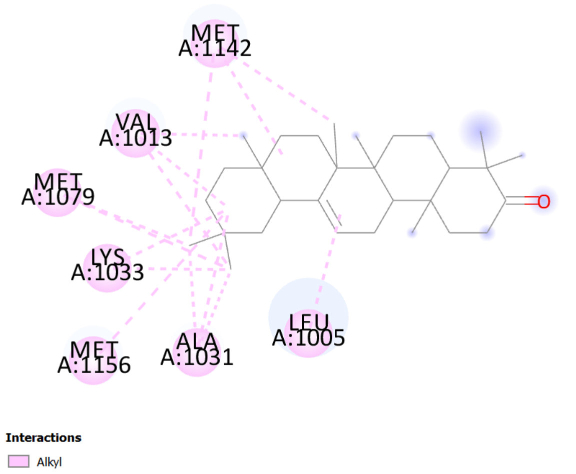

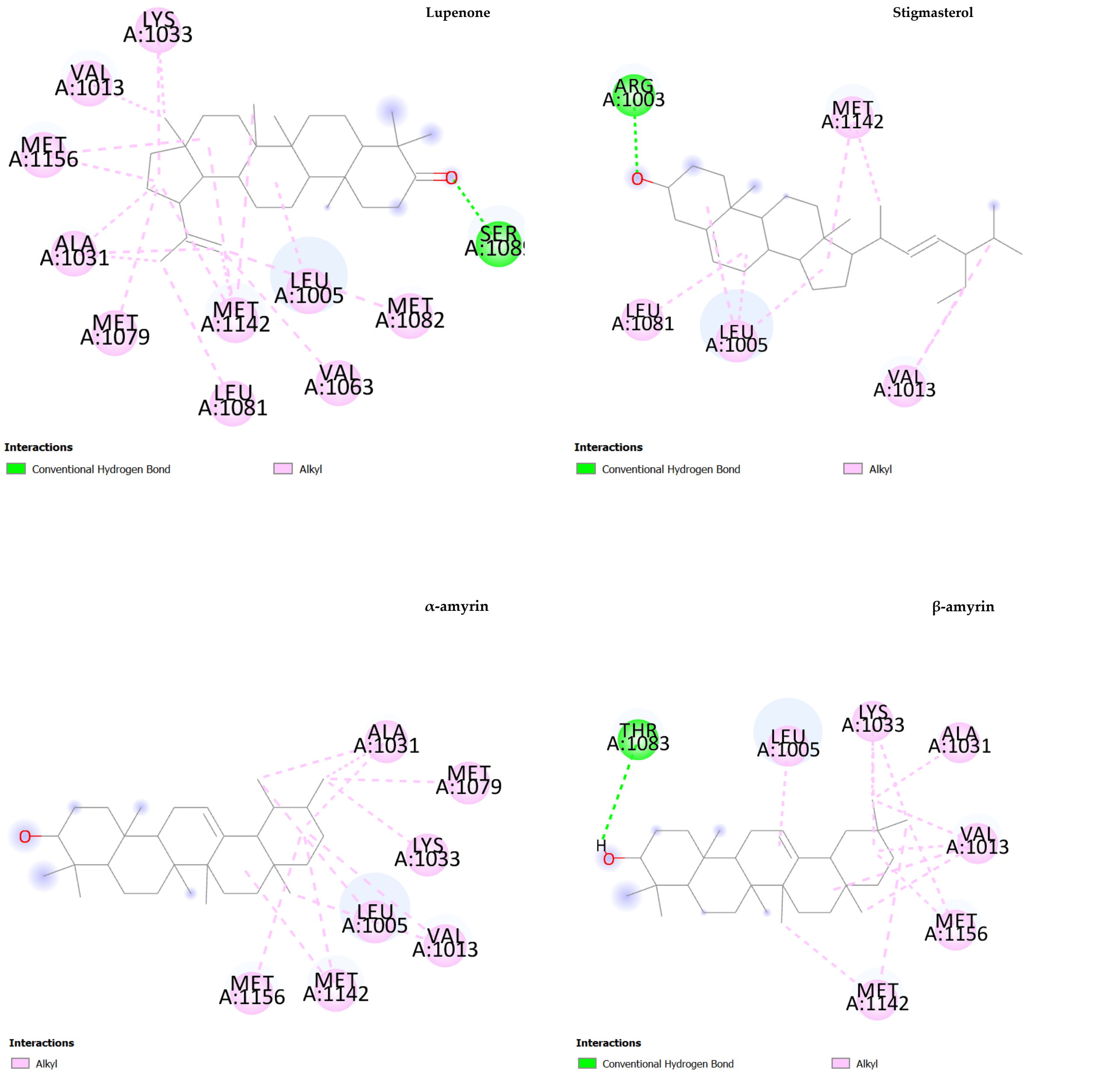

Figure S1. The results of the molecular docking study of IGFR were represented by 2D diagram of phytochemical-receptor interactions.

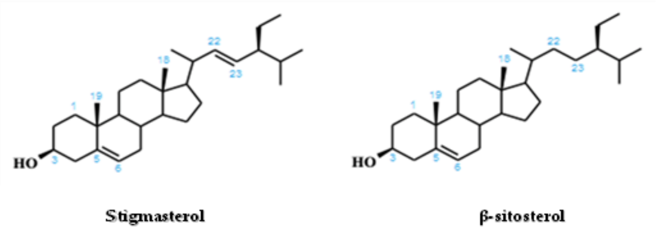

Figure S2. Structure of compound 1 (stigmasterol) and compound 2 (β-sitosterol) from fraction ACH3.

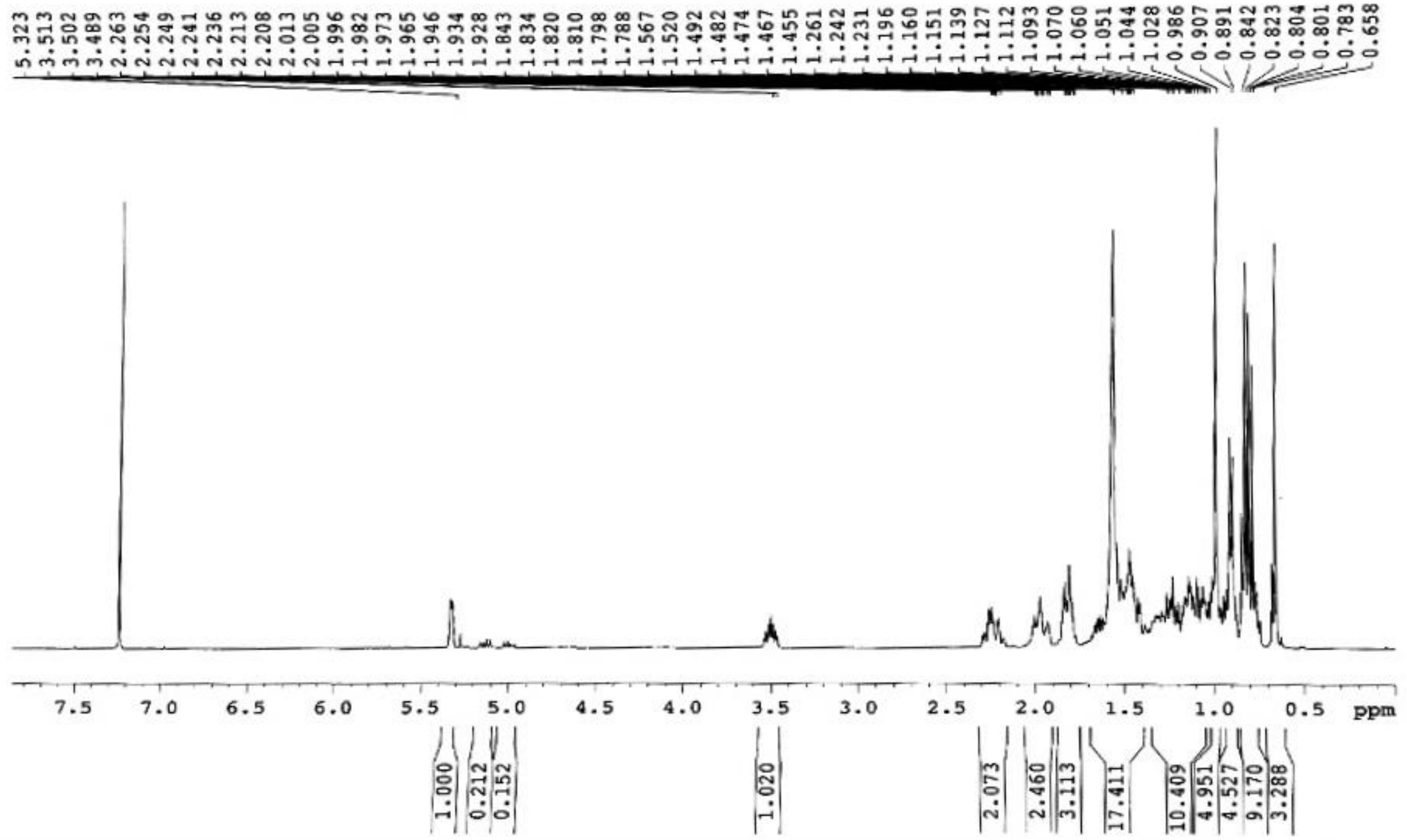

Figure S3. ^{1}H-NMR spectrum of a mixture of β-sitosterol and stigmastrol (compound 1 and 2) in $CDCl_3$

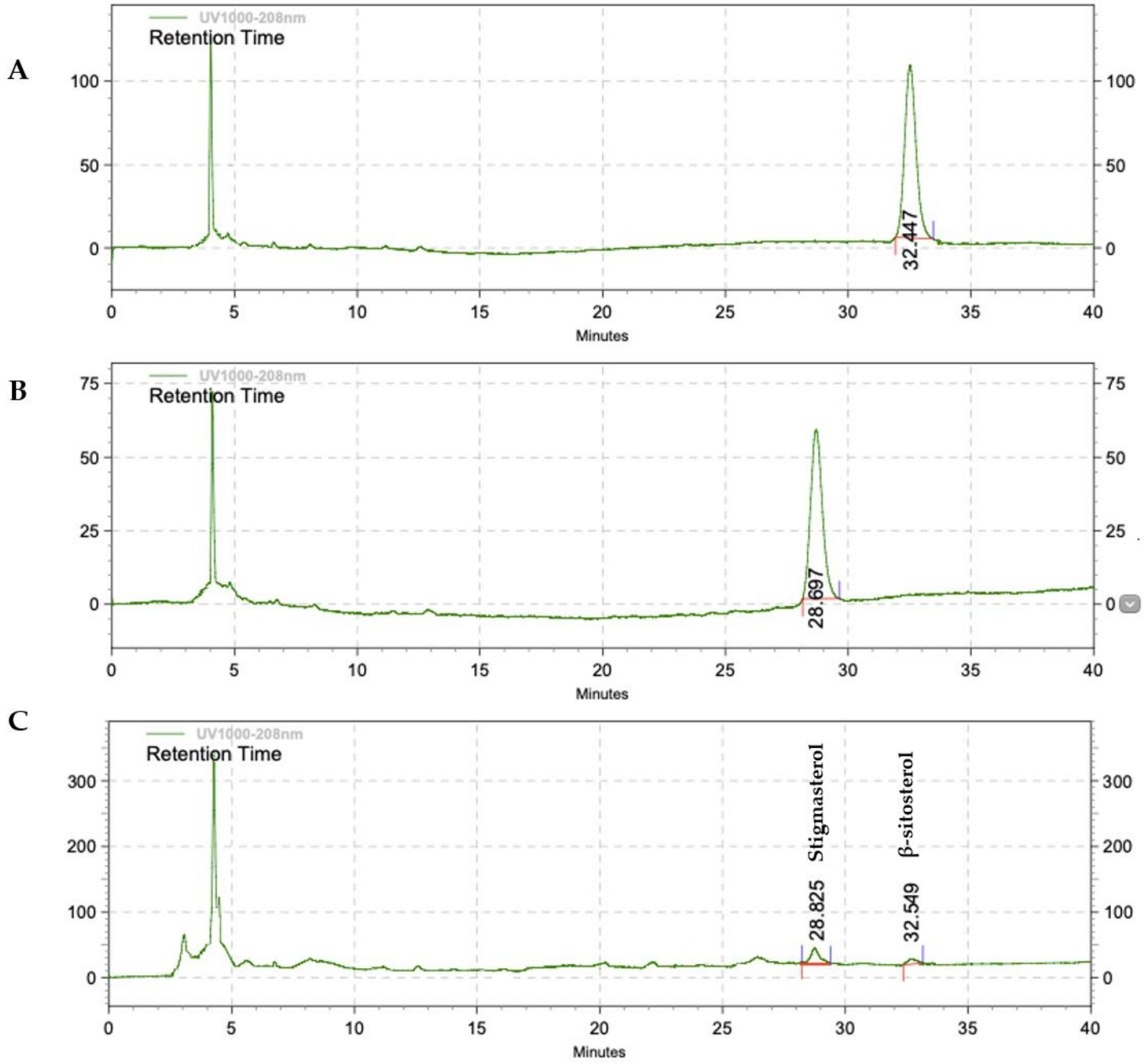

Figure S4. Reverse-phase HPLC analysis of standard β-sitosterol (A) and stigmasterol (B). The extract of ACH showing the presence of both β-sitosterol and stigmasterol (C).
